# Educational anxiety and student mental health in the era of artificial intelligence: a multi-source data fusion analysis in smart education

**DOI:** 10.3389/fpubh.2026.1804527

**Published:** 2026-05-26

**Authors:** Qing Tian

**Affiliations:** School of Art, Southeast University, Nanjing, China

**Keywords:** artificial intelligence, educational anxiety, intelligent education, student mental health, technology acceptance theory

## Abstract

**Introduction:**

Against the backdrop of the rapid advancement of educational digitalization and the growing prominence of student mental health issues, this study develops an integrated model of “technology perception–educational anxiety–student mental health” to examine the mechanisms through which perceived usefulness, perceived fairness, and perceived controllability are associated with student mental health.

**Methods:**

A cross-sectional questionnaire design was adopted, and data were collected from university students through a combination of online and offline surveys from July to September 2025, yielding 380 valid responses. Based on these quantitative data, structural equation modeling and artificial neural network techniques were employed to test the proposed hypotheses.

**Results:**

The results indicate that perceived usefulness, perceived fairness, and perceived controllability are all significantly and positively associated with student mental health, whereas educational anxiety is significantly and negatively associated with student mental health. All three dimensions of technology perception significantly reduce educational anxiety and indirectly promote student mental health through this mediating pathway, with perceived fairness and perceived controllability showing relatively stronger associations. Sensitivity analysis using the artificial neural network further reveals that perceived fairness is the most important predictor of educational anxiety, followed by perceived controllability and perceived usefulness.

**Discussion:**

This study enriches the theoretical understanding of student mental health in AI-enabled educational contexts and provides empirical implications for educational technology optimization, algorithm governance, and student mental health interventions.

## 1 Introduction

With the widespread penetration of artificial intelligence (AI) technologies into the field of education, applications such as intelligent homework grading (1), adaptive learning systems (2), learning analytics (3), and academic early warning platforms (4) are reshaping the fundamental forms of teaching, learning, and assessment. On the one hand, the advent of intelligent education provides unprecedented technological possibilities for enhancing teaching efficiency, optimizing learning pathways, and realizing personalized support (5); on the other hand, through new mechanisms such as algorithmic evaluation, data monitoring, and human–machine comparison, it reconstructs students' subjective perceptions of academic competition, fairness, and future development (6). This may trigger new types of educational anxiety and exert far-reaching impacts on students' mental health. How to strike a balance between “technological empowerment” and “psychological burden” has become an urgent issue that intelligent education practices must address.

Existing research has shown that academic pressure, competition for educational advancement, and uncertainty in educational outcomes are major sources of traditional educational anxiety (7), which is closely associated with mental health problems such as depression and reduced subjective wellbeing (8). However, as generative artificial intelligence becomes deeply embedded in course learning and formative assessment, the meaning of educational anxiety is undergoing a profound structural transformation. In contexts characterized by algorithmic opacity, intelligent judgment, and human–machine competition, students may increasingly experience feelings of being “monitored by data,” “defined by algorithms,” or even having their sense of agency supplanted (9). This form of “AI-induced educational anxiety” possesses a distinct theoretical core: it is neither equivalent to conventional academic or test anxiety nor reducible to general technostress. Traditional academic or test anxiety primarily arises from students' fear that their abilities are insufficient to meet established evaluation standards, whereas technostress generally refers to a sense of difficulty or helplessness at the operational level. By contrast, AI-induced educational anxiety stems from systemic uncertainty and a sense of existential threat—students worry about the opacity of evaluative standards, the possibility that their efforts may be easily discounted, and the loss of control in the face of intelligent systems.

Although there is a substantial body of literature on the effectiveness of AI applications in education, existing studies mainly focus on technology acceptance or performance evaluation of AI-assisted learning (10), and relatively few have explored in depth the negative spillover effects of intelligent technologies—as an environmental stressor—on students' mental health. Methodologically, prior research on educational anxiety and mental health has predominantly adopted linear regression or stand-alone structural equation modeling (SEM). Yet human psychological mechanisms are complex and subtle, and the process by which anxiety induces psychological problems often involves complex non-linear relationships that traditional linear statistical methods struggle to capture accurately, making it difficult to identify deeper patterns of interaction among variables or to conduct precise risk prediction for high-risk groups.

In response to these gaps, this study aims to systematically examine, within the context of intelligent education, the critical role played by subjective perceptions of AI in the relationship between educational anxiety and students' mental health. Specifically, at the theoretical level, this paper constructs a structural model of “subjective AI-related perceptions–educational anxiety–student mental health,” and employs a hybrid approach combining structural equation modeling (SEM) and artificial neural networks (ANN) for validation and extension: SEM is used for confirmatory analysis and path mechanism exploration, while ANN is used to uncover potential non-linear relationships and the relative importance of key predictors, thereby unifying theoretical explanation and predictive performance. On this basis, the study seeks to address the following research questions: (1) In the era of intelligent education, what is the relationship between students' subjective perceptions of AI in education and their level of educational anxiety? (2) Does AI-induced educational anxiety play a mediating role between subjective AI-related perceptions and students' mental health? If so, what are the specific pathways and effect sizes? (3) What similarities and differences emerge between SEM and ANN in depicting the relationship among “subjective AI-related perceptions–educational anxiety–mental health”? Can their complementary use enhance the explanatory power and predictive accuracy of the model, thereby providing more robust evidence for the risk governance of intelligent education?

## 2 Literature review and research hypotheses

### 2.1 Education anxiety triggered by artificial intelligence

As artificial intelligence technologies become progressively embedded in key educational processes such as teaching and learning, learning analytics, intelligent assessment, and personalized recommendation, students' psychological responses in educational settings have begun to exhibit new characteristics that go beyond traditional academic stress. Existing research indicates that student anxiety is typically closely associated with academic workload, exam-based evaluation, competition for further education, and uncertainty about the future, and that it significantly affects learning engagement, academic performance, and mental health (11). At the same time, the widespread application of information technology in education has also been found to induce technostress characterized by adaptation burden, cognitive overload, and the pressure of constant connectivity, thereby negatively influencing individuals' emotional states and behavioral performance (12, 13). However, artificial intelligence differs from ordinary digital tools: it not only serves as a means of learning support, but is also embedded in the educational process as an evaluator, predictor, and participant in decision-making. As a result, students are confronted not merely with the question of “whether they can use technology,” but with situations in which learning behaviors are tracked in real time, learning outcomes are visualized through algorithms, and individual abilities are modeled and predicted by intelligent systems. Under such conditions, students may easily develop feelings of being monitored, labeled, and deprived of control over the allocation of educational opportunities. In recent years, regarding emotional responses triggered by artificial intelligence and algorithmic systems, Dietvorst et al. (14) found that, compared with human judges, individuals are more likely to react strongly and negatively to algorithmic errors, thereby exhibiting a pronounced tendency toward algorithm aversion. Research in the field of education has likewise shown that when learning analytics systems, intelligent scoring, or automated feedback mechanisms lack transparency and interpretability, students may not only question the accuracy and fairness of their outcomes, but may also develop persistent psychological tension and defensive reactions (15).

Based on the aforementioned rationale, this study defines “educational anxiety triggered by AI” as a composite emotional experience distinct from technostress, academic anxiety, and test anxiety. Specifically, it refers to a state of persistent apprehension and tension experienced by students during AI's profound involvement in educational evaluation, feedback, and resource allocation, stemming from a sense of uncertainty regarding algorithmic fairness, system controllability, decision transparency, and their potential educational consequences. From the interdisciplinary perspective of educational psychology and technology governance, this conceptualization aids in further revealing that students' acceptance of educational AI relies not only on perceived functionality but is also profoundly influenced by institutional trust and a sense of emotional security.

### 2.2 Subjective perceptual characteristics of AI in education

A large body of educational technology research has demonstrated that “perceived usefulness” and “perceived ease of use” are key antecedents influencing learners' intention to use and their continued usage behavior (16, 17). Building on this, systematic reviews of AI in education indicate that students and teachers generally regard adaptive recommendation, personalized feedback, automated grading, and intelligent tutoring as the “core advantages” of AI systems, and they hold a high perception of these functions' potential to enhance learning efficiency and instructional support (18, 19). At the same time, compared with traditional educational technologies, learners are particularly sensitive to the “performance stability and predictive accuracy” of AI systems; once algorithmic feedback is perceived as “inaccurate” or “hard to interpret,” their overall perception of system effectiveness and their willingness to use it decline significantly (20).

In recent years, research has gradually shifted from a focus on “rational acceptance” to greater attention to emotional experience and human–machine relationships. On the one hand, variables such as technostress and computer self-efficacy have long been shown in early online learning and e-learning studies to significantly shape learners' subjective evaluation and acceptance of new technologies (21). In AI-enhanced educational environments, however, these affective variables have acquired new connotations: some learners experience discomfort and unease when “interacting with algorithms” or “being continuously monitored in their learning trajectories by the system,” which in turn reduces their trust in and reliance on the system (22). On the other hand, the emergence of generative large language models has intensified experiences of “anthropomorphized interaction” and “partner-like learning.” When interacting with conversational AI (e.g., ChatGPT), students tend to form anthropomorphic impressions regarding its “intelligence,” “reliability,” and “willingness to cooperate,” which differ markedly from the purely instrumental attributes of traditional educational software (23). Existing studies suggest that such perceptions of anthropomorphism and peer-likeness may not only enhance learning motivation and enjoyment, but also lead to overreliance and blurred responsibility, causing learners to experience role confusion between “self-directed learning” and “algorithmic agency (24).”

In sum, the subjective perceptual characteristics of AI in education are no longer reducible to a unidimensional stance of “acceptance” or “rejection,” but rather constitute a dynamic system encompassing expectations of cognitive empowerment, affective trust negotiations, and crises of ethical subjecthood. Current literature tends to focus on measuring single dimensions and lacks integrative investigations. Therefore, exploring how these perceptual characteristics interact and jointly influence educational effectiveness serves as the analytical entry point of this study.

### 2.3 Technology acceptance theory

The Technology Acceptance Model (TAM) is one of the most influential models for explaining individual technology adoption behavior. In the field of educational technology, a large number of empirical studies have employed TAM as an analytical framework to systematically examine the adoption of e-learning platforms, learning management systems, mobile learning, and online collaboration tools (25). King and He conducted a meta-analysis of TAM-related research and found that perceived usefulness and perceived ease of use exert stable and significant predictive effects on behavioral intention, and that the model's explanatory power is relatively consistent across different technologies and contexts (26). Focusing on online learning settings, Park, based on TAM, showed that university students' behavioral intention to use e-learning is primarily predicted jointly by perceived usefulness and self-regulated learning ability, whereas perceived ease of use influences intention mainly indirectly via perceived usefulness (21). Similarly, Šumak et al. (27) conducted a meta-analysis of e-learning technology acceptance studies and confirmed that TAM exhibits high robustness in explaining usage intentions across different user groups and types of online learning systems.

However, although existing studies on AI applications in education sometimes borrow TAM-related terminology (e.g., “perceived usefulness,” “performance expectancy”), they remain relatively fragmented at the model level and lack integrative attempts to systematically address “how AI-specific attributes reshape the acceptance process.” On the one hand, the systematic review by Zawacki-Richter et al. indicates that AI applications in higher education mainly revolve around automated grading, adaptive learning, and intelligent tutoring. Researchers predominantly rely on effectiveness indicators to evaluate outcomes, while paying insufficient attention to teachers' and students' subjective perceptions regarding fairness, responsibility, and the threat of replacement (18). On the other hand, in exploring the educational use of large models such as ChatGPT, Kasneci et al. (23) emphasize that students' acceptance of AI depends not only on its “intelligence” and “ease of use,” but is also shaped by their subjective judgments concerning academic integrity, creativity, and the meaning of learning.

Drawing on the above literature, this study attempts two innovative extensions of TAM. First, it expands “perceived usefulness” into a multidimensional construct encompassing perceived enhancement of academic performance, perceived improvement in learning efficiency, perceived promotion of competence development, and perceived increase in learning autonomy, so as to more precisely characterize the perceived utility of AI-based educational systems across different learning outcomes. Second, it incorporates AI-specific subjective perception dimensions such as “perceived fairness” and “perceived controllability,” and treats “educational anxiety” as a key emotional mediating variable linking subjective perception and behavioral intention. Through this extension, the study seeks to shift the focus of the classical acceptance framework from “whether to accept a given technology” to “how to understand and negotiate AI-based educational systems along the three dimensions of effectiveness, emotion, and ethics,” thereby providing a theoretical response to the increasingly salient mental health issues in current AI-in-education practice. Accordingly, the following research hypotheses are proposed:

H1: Perceived usefulness of AI positively affects students' mental health.

H2: Perceived fairness of AI positively affects students' mental health.

H3: Perceived controllability of AI positively affects students' mental health.

### 2.4 The impact of educational anxiety on students' mental health

With the rapid penetration of artificial intelligence into teaching, assessment, and educational governance, scholars at home and abroad have increasingly noted a phenomenon of “educational anxiety” characterized by a core sense of uncertainty, loss of control, and threat. Compared with traditional information technology anxiety, AI-induced anxiety arises not only from changes in tool operation and workload, but is also closely related to the redefinition of professional identity, the meaning of learning, and educational fairness (18, 28). At the student level, educational anxiety is more often manifested as multiple concerns about the meaning of learning, the rules of assessment, and privacy and security. Studies based on learning analytics suggest that although students generally acknowledge the value of data-based early warning and personalized support, worries about “being continuously monitored” and “being misjudged by algorithms” are equally salient: perceived privacy risk, perceived surveillance, and perceived unfairness significantly undermine their overall evaluation of such systems and are positively associated with levels of anxiety and stress (20).

In the wake of generative AI, on the one hand, when confronted with large models represented by ChatGPT, some students worry that their writing and thinking abilities will be “diluted” by technology and that their learning efforts will lose their meaning, thereby experiencing a sense of powerlessness associated with “being replaced in competence (29).” On the other hand, blurred boundaries of academic integrity make it difficult for students to grasp the line between “legitimate use” and “academic misconduct,” giving rise to persistent normative anxiety (23). In a study on ChatGPT and academic integrity, Cotton et al. found that, under the dual pressure of high-stakes assessment systems and easy access to AI tools, students tend to regard AI as a “shortcut” for coping with exam and assignment pressure, while simultaneously fearing being labeled as cheating, which significantly heightens anxiety related to assessment regimes (30).

**Figure 1:**
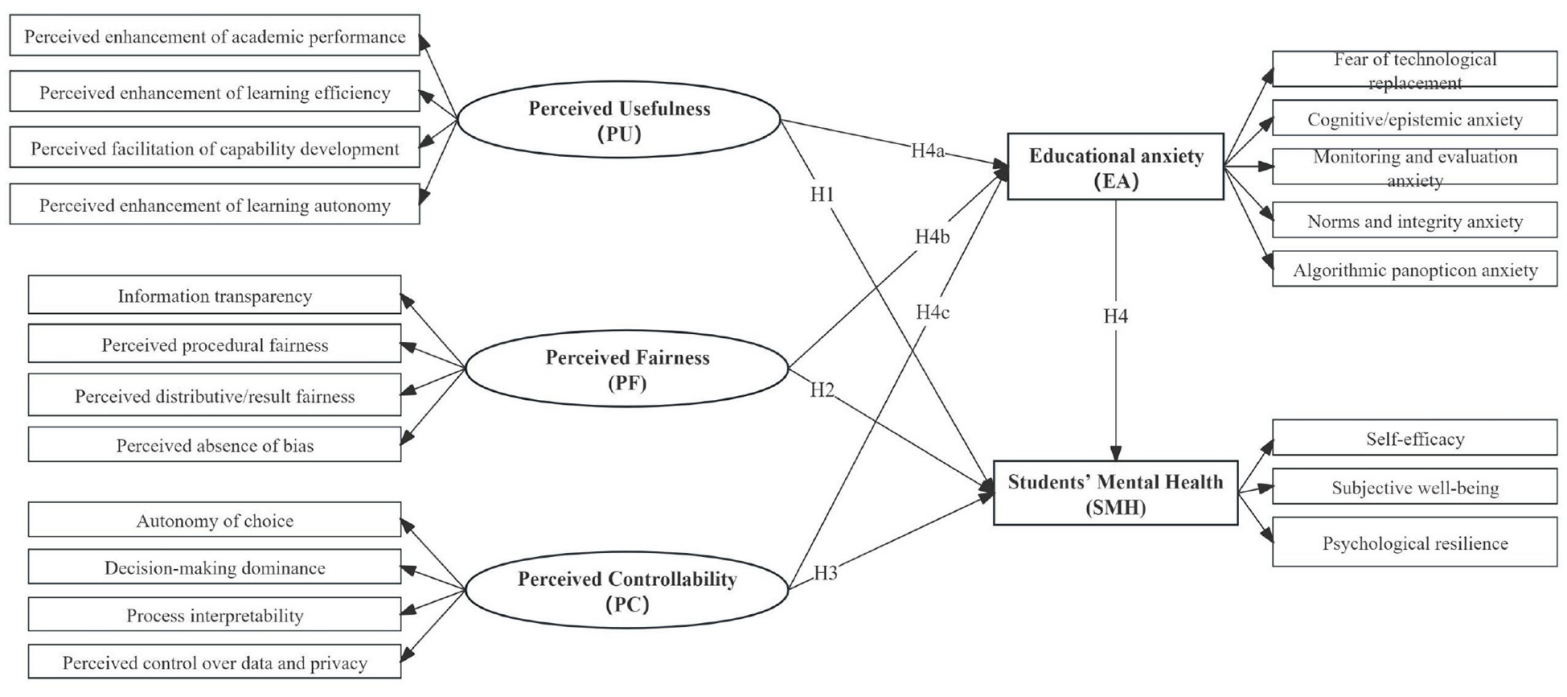
Hypothetical framework diagram.

On this basis, the research hypothesis model of this study is depicted in Figure 1. Building on a review of technostress and AI-in-education research, this study explores how different actors' subjective perceptions of AI influence students' mental health through educational anxiety. This integrative perspective helps move beyond treating anxiety merely as a single “negative emotion,” and instead conceptualizes it as a key mediating variable in the process of AI being re-embedded into the educational system, thereby providing a more explanatory theoretical anchor for subsequent empirical measurement and intervention design. Accordingly, the following research hypothesis H4 is proposed:

H4: Educational anxiety mediates the relationship between subjective AI-related perceptions in education and students' mental health.

H4a: Educational anxiety mediates the relationship between perceived usefulness of AI and students' mental health.

H4b: Educational anxiety mediates the relationship between perceived fairness of AI and students' mental health.

H4c: Educational anxiety mediates the relationship between perceived controllability of AI and students' mental health.

## 3 Research design and methodology

### 3.1 Research method and procedure design

This study adopts a two-stage, multi-method analytical framework combining covariance-based structural equation modeling (CB-SEM) and artificial neural networks (ANN) to systematically investigate the antecedents of university students' education anxiety in the context of artificial intelligence and the mechanism through which such anxiety affects students' mental health.

In the first stage, this study explicitly chooses covariance-based SEM (CB-SEM) rather than partial least squares SEM (PLS-SEM). Although PLS-SEM has advantages in handling non-normal data and complex models, the central objective of this study is to conduct a rigorous theoretical test and path confirmation of the extended Technology Acceptance Model (TAM), rather than merely exploratory prediction. CB-SEM offers irreplaceable statistical rigor in evaluating overall model fit and validating mediating mechanisms among latent constructs. To satisfy the strict assumptions required by CB-SEM, this study conducted a multivariate normality test using Mardia's coefficient prior to model estimation, thereby ensuring that the sample distribution met the requirements for maximum likelihood estimation. After establishing the reliability and validity of the measurement scales, model fit and modification were used to assess the path relationships among latent variables and to identify the linear causal mechanisms through which perceived usefulness, perceived fairness, and perceived controllability influence education anxiety and mental health.

**Figure 2:**
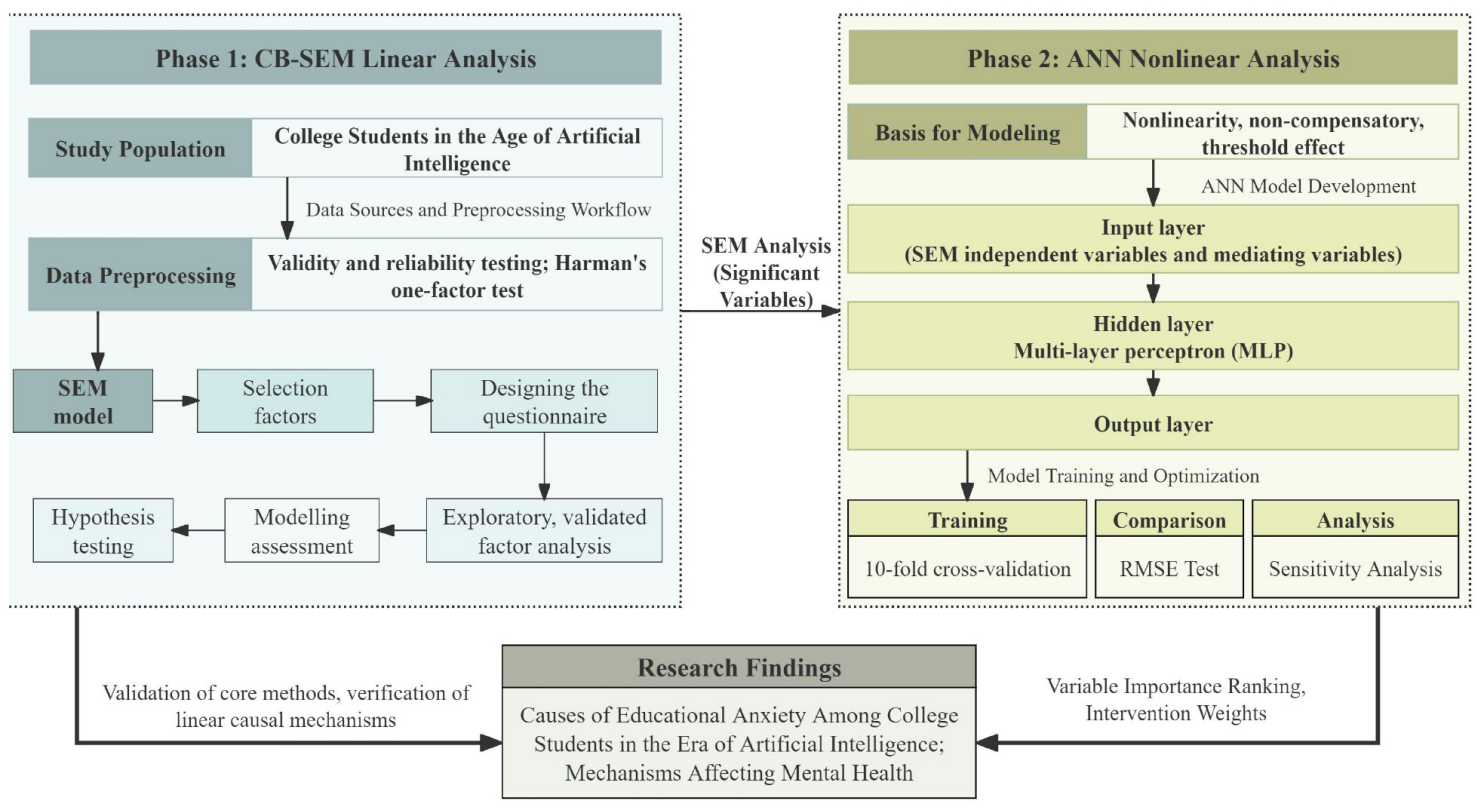
Research flowchart.

In the second stage, this study introduces artificial neural networks (ANN) for deeper analysis. It should be emphasized that the use of non-linear ANN is not merely a methodological supplement, but is grounded in a deeper theoretical necessity. Traditional SEM is based on linear assumptions, treating relationships among variables as symmetric and proportional. However, in the psychologically and cognitively charged environment shaped by artificial intelligence, students' psychological responses often exhibit non-compensatory and non-linear characteristics. For example, the damaging effect of “perceived unfairness” on students' mental health may be far greater than the positive utility generated by “perceived fairness.” Likewise, the negative effect of “education anxiety” on mental health may involve a threshold effect, whereby psychological defenses collapse precipitously once a critical point is exceeded. Such complex and asymmetric cognitive mechanisms are difficult for linear SEM to capture with precision. Therefore, this study employs a multilayer perceptron (MLP) to construct the ANN model, using as input-layer nodes the independent variables found to be significant in the SEM stage, with mental health as the output variable. To clearly demonstrate the incremental predictive advantage of ANN over SEM, the network was trained using 10-fold cross-validation, and the root mean square errors (RMSE) of the SEM and ANN models were rigorously compared across both training and testing datasets. Finally, sensitivity analysis was conducted to calculate the normalized relative importance of each predictor. This approach not only compensates for the limited explanatory power of SEM with respect to non-linear relationships, but also provides more precise weighting for policy interventions in the governance of artificial intelligence in educational settings. The methodological workflow described in this paper is shown in Figure 2.

### 3.2 Questionnaire structure and variable measurement

Before formally conducting the questionnaire survey, the research team developed a detailed research protocol specifying the study objectives, survey content, sampling scope, and data analysis methods. The protocol was subsequently submitted to the Science and Technology Ethics Committee of Southeast University for review and received ethical approval in July 2025 (Approval No. 2025-07), ensuring that the entire research process strictly complied with ethical standards and data protection requirements. In response to reviewers' concerns regarding methodological rigor, this study did not employ a strictly probabilistic cluster sampling approach; instead, it adopted a non-probability sampling strategy combining convenience sampling and snowball sampling. With the assistance of the research team's academic network and faculty members from several universities, questionnaire invitations were distributed to target students, clearly explaining the study purpose, participation procedures, and commitments to data confidentiality, thereby safeguarding participants' rights to informed consent and privacy.

**Figure 3:**
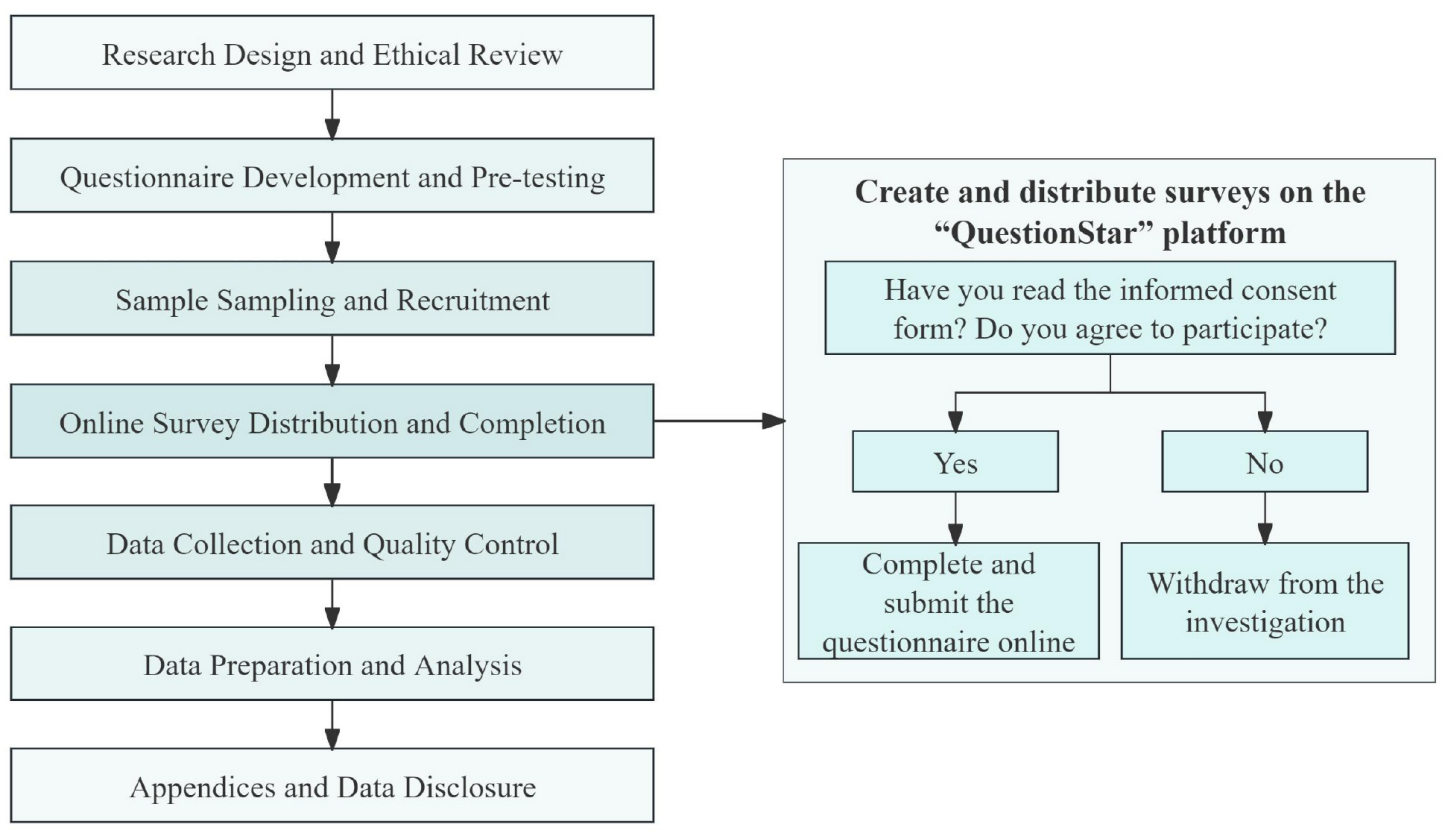
Procedural steps for questionnaire distribution.

During the formal survey stage, the research team designed and distributed the online questionnaire via the Wenjuanxing platform. Participants could access the questionnaire page by clicking a link or scanning a QR code on a mobile device or computer. Prior to completing the questionnaire, all participants were required to read and agree to the online informed consent form, confirming their voluntary participation in the study. Throughout the data collection period, the team monitored the response progress in real time and regularly checked the completeness and consistency of the questionnaires. Responses with excessively short completion times, obviously inconsistent answers, or logical errors were excluded to ensure data validity and reliability. The specific questionnaire distribution procedure is shown in Figure 3.

This study employed a structured questionnaire for data collection. The questionnaire consisted primarily of two parts: (1) respondents' demographic information, such as gender, age, academic year, and major; and (2) measurement items for the core variables of the study. All measurement items were rated on a five-point Likert scale (1 = “strongly disagree,” 5 = “strongly agree”). The specific items are presented in Table 2. To ensure content validity and measurement appropriateness, the research team organized an expert review prior to the formal distribution of the questionnaire. A total of five experts were invited to evaluate the instrument, including two professors in the field of education, two professors in the field of psychology, and one researcher specializing in AI in education. All of these experts had relevant research experience or experience in questionnaire design. The expert review focused primarily on the consistency between the items and the theoretical constructs, the accuracy and clarity of the wording, the comprehensibility of the questionnaire language, and the degree to which the measurement content aligned with the context of AI applications in education. Based on the experts' feedback, the research team made targeted revisions to several measurement items, adjusting the wording of individual items to reduce ambiguity, removing expressions with semantic overlap or unclear meaning, standardizing the semantic direction of some items, and strengthening the correspondence between the items and AI-based educational scenarios. The revised version was then finalized as the formal questionnaire.

### 3.3 Scale development and measurement indicators

In this study, the scale is divided into five first-order dimensions: perceived usefulness, perceived fairness, perceived controllability, educational anxiety, and students' mental health (Table 1). Specifically, the perceived usefulness dimension measures respondents' subjective evaluations of AI in four aspects: “perceived improvement in academic performance,” “perceived improvement in learning efficiency,” “perceived facilitation of competence development,” and “perceived enhancement of learning autonomy.” These items assess how respondents view AI in terms of improving objective performance and output quality, saving time and effort, promoting higher-order capability development, and supporting personalized and self-directed learning. The perceived fairness dimension consists of four second-order variables: “information transparency,” “perceived procedural fairness,” “perceived distributive/result fairness,” and “perceived absence of bias.” It is used to examine whether students are aware of how data are collected and used, whether they regard algorithmic decision-making processes as consistent and transparent, whether they consider AI-generated evaluations to be accurate and just, and whether the system avoids discriminatory outputs based on gender, race, or background. The perceived controllability dimension, through four aspects—”autonomy of choice,” “decision-making dominance,” “process interpretability,” and “perceived control over data and privacy”—assesses the extent to which students believe they can independently decide whether to use AI, maintain a leading role in critical learning decisions, understand the logic behind AI decisions, and retain control over the collection, storage, and deletion of their personal data. The educational anxiety dimension is delineated from several perspectives, including “fear of technological replacement,” “cognitive/epistemic anxiety,” “monitoring and evaluation anxiety,” “norms and integrity anxiety,” and “algorithmic panopticon anxiety.” It captures students' feelings of unease and stress associated with concerns about being replaced in future careers, difficulties in distinguishing the authenticity of knowledge, the risk of being improperly monitored or evaluated by AI, the possibility of violating academic integrity norms, and the pressure of continuous data tracking. The students' mental health dimension includes three components: “self-efficacy,” “subjective wellbeing,” and “psychological resilience.” These respectively evaluate students' level of confidence when facing challenging tasks, their sense of pleasure and achievement in the learning process, and their capacity to recover and adjust in the face of academic setbacks and technological change.

**Table 1:** Questionnaire design.

*First-level dimension*	Second-level explanatory variable	Questionnaire question
*Perceived Usefulness (PU)*	Perceived enhancement of academic performance	Whether AI helps users improve objective grades, quality of output, or work efficiency.
	Perceived enhancement of learning efficiency	Whether AI helps users complete the same or a larger amount of learning tasks with less time and effort.
	Perceived facilitation of capability development	Whether AI helps users understand complex concepts, cultivate critical thinking, or acquire long-term skills.
	Perceived enhancement of learning autonomy	Whether AI enables users to customize their learning pathways and control their learning progress according to their own pace.
	Information transparency	Whether users are informed about how their data are collected and used.
*Perceived Fairness (PF)*	Perceived procedural fairness	Whether the decision logic of AI algorithms is consistent and transparent, and whether the evaluation criteria treat all users equally.
	Perceived distributive/result fairness	Whether the evaluation results provided by AI accurately reflect users' actual efforts and competence.
	Perceived absence of bias	Whether users believe that the AI system avoids discriminatory recommendations or evaluations based on gender, race, or specific backgrounds.
*Perceived Controllability (PC)*	Autonomy of choice	Whether users have the right to decide “when to use and when to turn off AI,” rather than being forced to accept it.
	Decision-making dominance	In human–AI collaboration, whether users perceive themselves (rather than the algorithm) as the ultimate bearer of responsibility and the final decision-maker.
	Process interpretability	Whether users are able to understand the reasons behind specific decisions made by AI.
	Perceived control over data and privacy	The extent to which users perceive that they have the ability to manage, withdraw, or delete personal privacy data collected by the AI system.
	Fear of technological replacement	Worry that AI will replace occupations, rendering students' future skills worthless (crisis of professional identity).
	Cognitive/epistemic anxiety	Worry about being unable to distinguish the authenticity of AI-generated information, leading to uncertainty in knowledge acquisition.
*Educational Anxiety (EA)*	Monitoring and evaluation anxiety	Worry that AI may misinterpret learning behaviors or issue unjust negative evaluations, thereby affecting academic records.
	Norms and integrity anxiety	Worry that, when using AI to assist learning, one may unintentionally violate rules of academic integrity due to blurred boundaries.
	Algorithmic panopticon anxiety	Worry that one's learning behaviors are continuously recorded, analyzed, and evaluated by AI, resulting in excessive psychological pressure.
*Students' Mental Health (SMH)*	Self-efficacy	When facing new learning tasks, even if the content is very difficult, believing that one can find appropriate ways to complete them.
	Subjective wellbeing	Overall, often feeling pleasure and a sense of achievement during the learning process.
	Psychological resilience	The ability to actively adjust, recover, and remain engaged in learning when confronted with academic difficulties, technological change, or failure.

### 3.4 Data collection and sample characteristics analysis

This study targeted university students as the primary survey population and employed a questionnaire-based method for data collection. Questionnaire distribution adopted a combined online and offline approach. For the online strategy, questionnaire links were disseminated through education-related social media platforms as well as various student academic and social communication groups, and respondents were encouraged to forward the questionnaire to eligible peers. For the offline strategy, after obtaining permission from relevant university instructors or campus administrators, members of the research team distributed paper invitations containing the Wenjuanxing link or QR code to students in locations such as after public elective classes, in university library study areas, and at student club activity venues, inviting them to scan the code and complete the questionnaire on site. This multi-channel sampling and distribution approach helped maximize coverage of students from different universities and academic backgrounds. Data were collected from July 2025 to September 2025. During this period, a total of 450 questionnaires were distributed, of which 380 valid responses were recovered, yielding an effective response rate of 84.4%. To ensure sample quality, all returned questionnaires were rigorously screened, and invalid or logically inconsistent responses were excluded, thereby ensuring the reliability of the data and the validity of the study findings.

Regarding sample characteristics, female respondents accounted for 62.4% of the sample, while male respondents accounted for 37.6%. The age distribution was concentrated primarily between 18 and 22 years old, representing 68.4% of the sample, while those aged 23 to 25 accounted for 24.7%, and the remainder were aged 26 or above. Academic levels ranged from first-year undergraduates to fourth-year undergraduates and postgraduate students, with second-year and third-year students comprising relatively larger proportions, at 29.2% and 27.6%, respectively. Respondents represented a wide range of disciplines, including science and engineering, humanities, arts, management, and other fields, thereby ensuring sample diversity and representativeness. In addition, the respondents were drawn from multiple universities across different regions, enabling the study to more comprehensively reflect the current state of educational anxiety and mental health among university students in the context of the widespread application of artificial intelligence technologies.

## 4 Structural equation modeling analysis

### 4.1 Reliability and validity analysis of the scales

As shown in Table 2, this study examined the reliability and convergent validity of each latent variable. Specifically, the Cronbach's α coefficients for all latent variables were greater than 0.8, indicating good internal consistency reliability of the scales. The Cronbach's α values for perceived usefulness, perceived fairness, perceived controllability, educational anxiety, and students' mental health were 0.802, 0.820, 0.839, 0.841, and 0.843, respectively, all exceeding the recommended threshold of 0.7. With regard to composite reliability (CR), the CR values of all latent variables ranged from 0.803 to 0.844, all above 0.7, further confirming the reliability of the scales. In terms of convergent validity, the average variance extracted (AVE) for each latent variable was greater than 0.5-−0.508, 0.540, 0.568, 0.516, and 0.643, respectively—indicating satisfactory convergent validity. In addition, all standardized factor loadings for the measurement items were above 0.6, suggesting that each item adequately reflects its corresponding latent construct. Taken together, the measurement instruments for the latent variables used in this study exhibit good reliability and convergent validity, and are suitable for subsequent structural equation modeling analysis.

**Table 2:** Reliability and convergent validity tests for each latent variable.

Construct	Item	Cronbach's α	CR	AVE
PU	PU1	0.802	0.803	0.508
	PU2			
	PU3			
	PU4			
PF	PF1	0.820	0.823	0.540
	PF2			
	PF3			
	PF4			
PC	PC1	0.839	0.840	0.568
	PC2			
	PC3			
	PC4			
EA	EA1	0.841	0.842	0.516
	EA2			
	EA3			
	EA4			
	EA5			
SMH	SMH1	0.843	0.844	0.643
	SMH2			
	SMH3			

PU, perceived usefulness; PF, perceived fairness; PC, perceived controllability; EA, educational anxiety; SMH, student mental health.

To assess whether the sample data were suitable for factor analysis, this study conducted a Kaiser–Meyer–Olkin (KMO) measure of sampling adequacy and Bartlett's test of sphericity. As shown in Table 3, the KMO value is 0.888, well above 0.8, indicating that the sample data are appropriate for factor analysis. Bartlett's test of sphericity yielded a chi-square value of 3,657.334 with 190 degrees of freedom and a *p*-value of 0.000, reaching a significant level (*p* <0.001), further demonstrating that there are strong correlations among the variables and that the data are suitable for subsequent factor analysis.

**Table 3:** KMO and Bartlett's sphericity test results.

KMO		0.888
Bartlett's sphericity	spherical test	3,657.334
	*df*-value	190
	*p*-value	0.000

In addition, because this study relied on self-reported questionnaire data collected from the same source at a single point in time, there is a potential risk of common method bias (CMB). To assess this issue, Harman's single-factor test was employed, in which all measurement items corresponding to the latent variables were entered into an unrotated exploratory factor analysis. The results indicated that the first common factor accounted for 39.98% of the total variance, which did not exceed the empirical threshold of 50% (31). Therefore, it can be concluded that serious common method bias is not a concern in this study.

### 4.2 Assessment of discriminant validity

As shown in Table 4, the discriminant validity of each latent variable was evaluated using the Fornell–Larcker criterion. The square root of the average variance extracted (AVE) for each construct is presented in bold on the diagonal, whereas the off-diagonal elements represent the correlation coefficients between constructs (32). The results indicate that, for each construct, the square root of its AVE is greater than its correlations with other constructs. Specifically, the square roots of the AVE values for students' mental health (SMH), educational anxiety (EA), perceived controllability (PC), perceived fairness (PF), and perceived usefulness (PU) are 0.831, 0.693, 0.934, 0.597, and 0.427, respectively. All of these values exceed the corresponding inter-construct correlation coefficients in the same rows and columns. These findings demonstrate that each construct in this study exhibits good discriminant validity and is empirically distinct from the others.

**Table 4:** Discriminant validity test results (Fornell-Larcker Criteria).

	SMH	EA	PC	PF	PU
SMH	**0.831**				
EA	−0.481	**0.693**			
PC	0.487	−0.375	**0.934**		
PF	0.454	−0.334	0.309	**0.597**	
PU	0.380	−0.285	0.300	0.319	**0.427**

1. The on-diagonal items in bold represent the square roots of the AVE; 2. off-diagonal elements are the correlation; 3. PU, Perceived Usefulness; PF, perceived fairness; PC, perceived controllability; EA, educational anxiety; SMH, student mental health.

Table 5 reports the results of the discriminant validity assessment using the heterotrait–monotrait (HTMT) ratio. The table presents the HTMT values for all pairs of constructs. According to widely accepted threshold criteria, HTMT values should be below 0.85 to indicate adequate discriminant validity (33). The HTMT values among all constructs are lower than the recommended threshold, suggesting that each construct is empirically distinct from the others, thereby further confirming the discriminant validity of the measurement model.

**Table 5:** Discriminant validity test results (HTMT ratio).

	SMH	EA	PC	PF	PU
SMH					
EA	−0.633				
PC	0.553	−0.467			
PF	0.645	−0.520	0.413		
PU	0.638	−0.524	0.475	0.632	

PU, perceived usefulness; PF, perceived fairness; PC, perceived controllability; EA, educational anxiety; SMH, student mental health.

### 4.3 Results of confirmatory factor analysis (CFA)

Table 6 presents the fit indices of the confirmatory factor analysis (CFA) model used in this study. The results show that the CMIN/DF value is 2.731, which falls within the acceptable range of 1 to 3, indicating a good model fit. The GFI (0.899) and AGFI (0.867) are both close to or above 0.8, suggesting satisfactory model fit. The RMSEA is 0.068, below the 0.08 threshold, further supporting the adequacy of the model. In addition, the IFI (0.922), TLI (0.907), and CFI (0.922) are all greater than 0.9, while the NFI is 0.883 (above 0.8). These indices collectively indicate an excellent overall model fit. In summary, all major fit indices reach or approximate the recommended criteria, demonstrating that the measurement model adopted in this study exhibits good structural adequacy and explanatory power.

**Table 6:** Validated factor analysis model fit.

Fitting index	Acceptable range	Measured value
CMIN		436.961
DF		160
CMIN/DF	1–3	2.731
GFI	≥0.8	0.899
AGFI	≥0.8	0.867
RMSEA	<0.08	0.068
IFI	≥0.9	0.922
NFI	≥0.8	0.883
TLI(NNFI)	≥0.9	0.907
CFI	≥0.9	0.922

### 4.4 Path coefficients of the structural model and hypothesis testing

**Figure 4:**
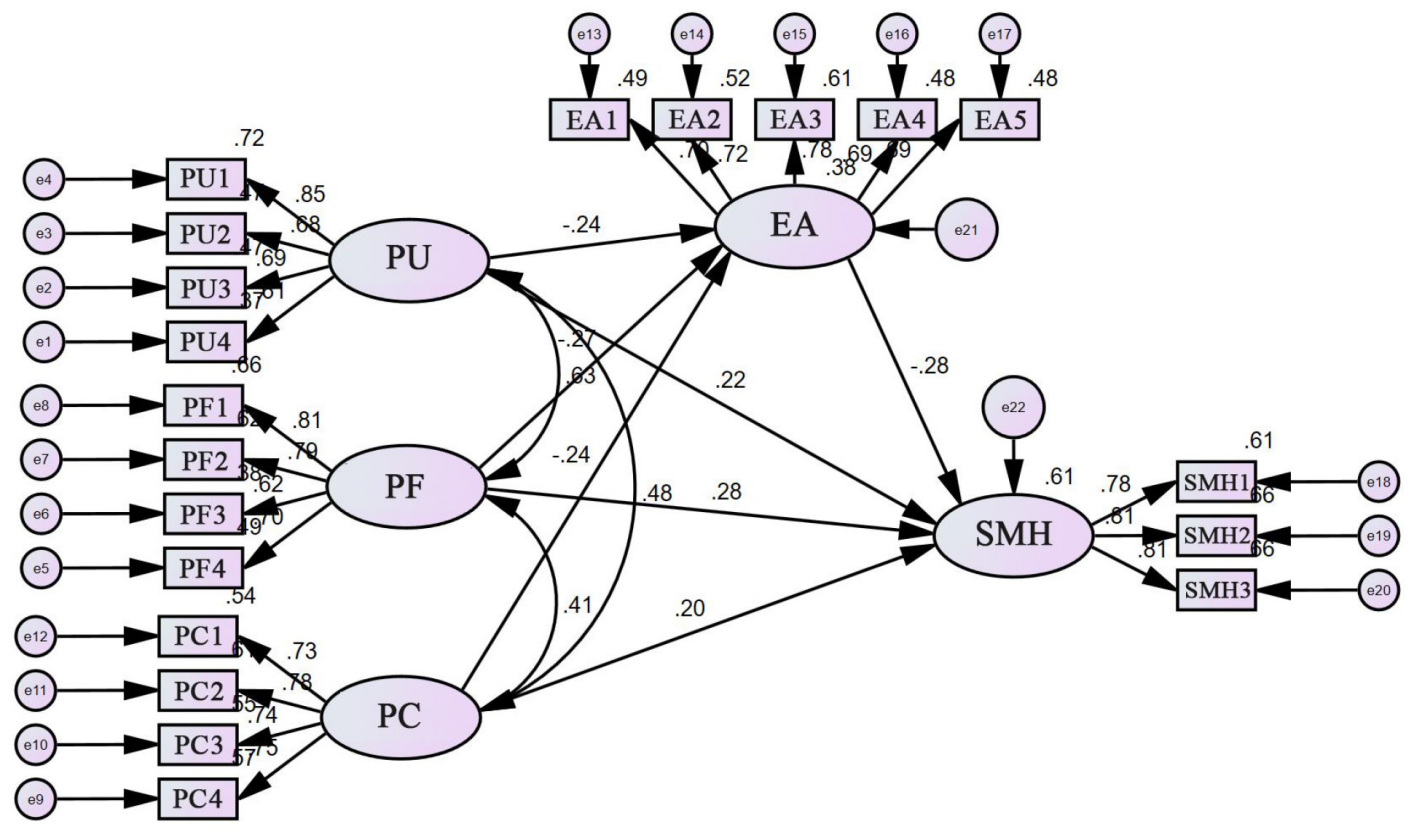
Path diagram of the structural equation model.

As shown in Table 7 and Figure 4, perceived usefulness (PU), perceived fairness (PF), and perceived controllability (PC) all exert significant positive effects on students' mental health (SMH), with path coefficients of 0.306, 0.326, and 0.193, respectively (all *p*-values <0.01). In contrast, educational anxiety (EA) has a significant negative effect on students' mental health (path coefficient = −0.306, *p* <0.001). Moreover, perceived usefulness, perceived fairness, and perceived controllability all show significant negative effects on educational anxiety, with path coefficients of −0.304, −0.290, and −0.209, respectively (all *p*-values <0.01). All hypothesized paths are supported by the data, indicating that perceived usefulness, fairness, and controllability not only directly influence students' mental health, but also indirectly promote better mental health by reducing educational anxiety.

**Table 7:** Path coefficients of structural model and hypothesis testing results.

Hypothesis				Estimate	S.E.	C.R.	*P*	Testing the hypothesis
H1	SMH	<–	PU	0.306	0.099	3.082	0.002	Established
H2	SMH	<–	PF	0.326	0.081	4.017	^***^	Established
H3	SMH	<–	PC	0.193	0.053	3.610	^***^	Established
H4	SMH	<–	EA	−0.306	0.069	−4.401	^***^	Established
H4a	EA	<–	PU	−0.304	0.104	−2.928	0.003	Established
H4b	EA	<–	PF	−0.290	0.083	−3.497	^***^	Established
H4c	EA	<–	PC	−0.209	0.056	−3.724	^***^	Established

^***^indicates *p* <0.001; PU: perceived usefulness; PF: perceived fairness; PC: perceived controllability; EA: educational anxiety; SMH: student mental health.

### 4.5 Mediation analysis

Table 8 presents the test results for the mediating effects of educational anxiety (EA) on the relationships between perceived usefulness (PU), perceived fairness (PF), perceived controllability (PC), and students' mental health (SMH). The results show that, for all three mediating paths, the indirect effects, direct effects, and total effects are statistically significant. Specifically, the indirect effects of PU, PF, and PC on SMH via EA are 0.093 (*p* = 0.005), 0.089 (*p* = 0.010), and 0.064 (*p* = 0.002), respectively, with confidence intervals that do not include zero. This indicates that educational anxiety plays a partial mediating role in the relationships between these three perceptual constructs and students' mental health. At the same time, the direct effects and total effects of PU, PF, and PC on SMH are also significant, further suggesting that perceived usefulness, fairness, and controllability not only exert direct influences on students' mental health, but also indirectly enhance students' mental health by reducing educational anxiety.

**Table 8:** Results of mediating effect tests.

Mediation path	Effect	Estimate	Lower	Upper	*P*
PU—EA—SMH	Indirect Effect	0.093	0.021	0.202	0.005
	Direct Effect	0.306	0.047	0.555	0.024
	Total Effect	0.399	0.148	0.648	0.005
PF—EA—SMH	Indirect Effect	0.089	0.018	0.185	0.010
	Direct Effect	0.326	0.126	0.546	0.001
	Total Effect	0.415	0.213	0.642	0.001
PC—EA—SMH	Indirect Effect	0.064	0.019	0.136	0.002
	Direct Effect	0.193	0.054	0.330	0.005
	Total Effect	0.256	0.121	0.395	0.001

PU, perceived usefulness; PF, perceived fairness; PC, perceived controllability; EA, educational anxiety; SMH, student mental health.

## 5 Analysis of ANN-based neural network models

**Figure 5:**
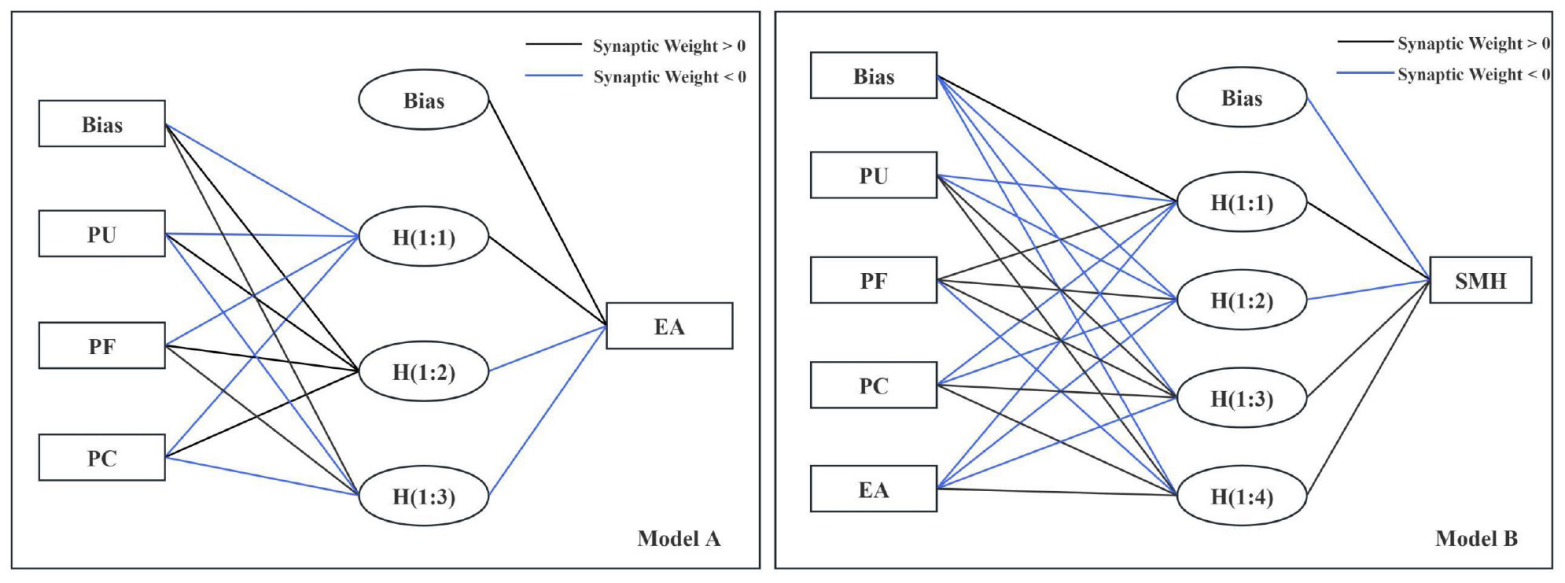
Artificial neural network model construction.

To further examine potential non-linear relationships among variables and improve the predictive accuracy of the outcome variables, this study introduced an artificial neural network (ANN) approach on the basis of structural equation modeling (SEM), thereby constructing an SEM–ANN integrated prediction model for student mental health. Specifically, based on the key antecedent variables identified through the significant paths in the SEM analysis, two feedforward backpropagation neural network models were further developed (Figure 5). In Model A, perceived usefulness (PU), perceived fairness (PF), and perceived controllability (PC) were used as input variables, with education anxiety (EA) as the output variable. In Model B, perceived usefulness (PU), perceived fairness (PF), perceived controllability (PC), and education anxiety (EA) served as input variables, while student mental health (SMH) was the output variable. This modeling strategy helps capture potentially complex non-linear patterns on the basis of validated theoretical path relationships.

With respect to dataset partitioning, to avoid model overfitting and ensure objective evaluation, a 10-fold cross-validation strategy was adopted, with the research sample divided into a training set (70%) and a testing set (30%). The training set was used for model parameter learning, whereas the testing set was used to assess the model's generalization ability. Both models were trained using a multilayer perceptron (MLP) architecture and the backpropagation algorithm, with a single hidden layer. Based on the number of input variables and model fitting performance, Models A, B, and C were each configured with a corresponding number of neurons in the hidden layer. In terms of activation functions, the Sigmoid function was used in the hidden layer and a linear function in the output layer. During training, mean squared error (MSE) was used as the loss evaluation metric, and the model's stability and predictive accuracy were assessed in conjunction with the testing set results. By comparing the error performance of the training and testing sets, the study further evaluated the supplementary predictive value of the ANN model relative to the linear structural model.

### 5.1 Root mean square error (RMSE) test

Table 9 presents the root mean square error (RMSE) test results for the two artificial neural network models (Model A and Model B) on the training and test sets. Model A uses perceived usefulness (PU), perceived fairness (PF), and perceived controllability (PC) as inputs to predict educational anxiety (EA), whereas Model B uses PU, PF, PC, and educational anxiety (EA) as inputs to predict students' mental health (SMH). Across 10 neural network runs, the mean RMSE values on the training and test sets were 0.357 and 0.306 for Model A, and 0.260 and 0.231 for Model B, respectively, with relatively small standard deviations, indicating good stability and generalization ability of the models. Overall, the RMSE of Model B in predicting students' mental health is slightly lower than that of Model A in predicting educational anxiety, suggesting that Model B exhibits higher predictive accuracy.

**Table 9:** Root mean square error test for artificial neural network models.

Neural network	Model A		Model B	
	Input: PU, PF, PC		Input: PU, PF, PC, EA	
	Output: EA		Output: SMH	
	Training	Testing	Training	Testing
ANN1	0.354	0.261	0.258	0.289
ANN2	0.349	0.191	0.266	0.253
ANN3	0.355	0.188	0.265	0.281
ANN4	0.358	0.200	0.256	0.220
ANN5	0.343	0.374	0.259	0.182
ANN6	0.360	0.310	0.264	0.149
ANN7	0.363	0.368	0.233	0.348
ANN8	0.348	0.423	0.272	0.181
ANN9	0.357	0.309	0.265	0.174
ANN10	0.379	0.389	0.259	0.237
Mean	0.357	0.306	0.260	0.231
SD	0.102	0.303	0.103	0.250

PU, perceived usefulness; PF, perceived fairness; PC, perceived controllability; EA, educational anxiety; SMH, student mental health.

### 5.2 Variable sensitivity analysis

Table 10 presents the sensitivity analysis results for ANN Model A and Model B. By evaluating the relative importance of each input variable, the extent to which different variables affect the model's prediction outcomes can be revealed. In Model A, perceived fairness (PF) has the highest mean relative importance (0.367), with a normalized relative importance of 100%, followed by perceived controllability (PC, 0.345, 94.01%) and perceived usefulness (PU, 0.288, 78.47%). This indicates that, in predicting educational anxiety, perceived fairness and perceived controllability play a more critical role.

**Table 10:** Analysis of the importance of normalization in artificial neural network models.

Neural network	Model A (Output: EA)			Model B (Output: TW)			
	PU	PF	PC	PU	PF	PC	EA
ANN1	0.276	0.409	0.315	0.364	0.190	0.294	0.152
ANN2	0.254	0.400	0.346	0.199	0.373	0.148	0.280
ANN3	0.315	0.384	0.301	0.221	0.283	0.205	0.290
ANN4	0.306	0.290	0.404	0.300	0.227	0.189	0.284
ANN5	0.320	0.350	0.330	0.250	0.220	0.212	0.318
ANN6	0.339	0.229	0.432	0.257	0.290	0.142	0.311
ANN7	0.028	0.464	0.508	0.216	0.278	0.213	0.294
ANN8	0.275	0.435	0.290	0.205	0.221	0.297	0.277
ANN9	0.229	0.362	0.409	0.234	0.243	0.184	0.339
ANN10	0.537	0.344	0.119	0.257	0.220	0.169	0.354
Average relative imporance	0.288	0.367	0.345	0.250	0.255	0.205	0.290
Normanlized relative importance (%)	78.474	100.000	94.005	86.207	87.931	70.690	100.000

PU, perceived usefulness; PF, perceived fairness; PC, perceived controllability; EA, educational anxiety; SMH, student mental health.

In Model B, educational anxiety (EA) exhibits the highest mean relative importance (0.290, 100%), followed by perceived fairness (PF, 0.255, 87.93%), perceived usefulness (PU, 0.250, 86.21%), and perceived controllability (PC, 0.205, 70.69%). This suggests that, in predicting students' mental health, educational anxiety is the most influential factor, followed by perceived usefulness and perceived fairness.

## 6 Discussion and conclusion

### 6.1 Discussion

Based on the integrated analysis of structural equation modeling (SEM) and artificial neural networks (ANN), this study found significant associations between perceived usefulness, perceived fairness, perceived controllability, and university students' education anxiety and mental health. Overall, the results support the following relational pattern: when students are more likely to perceive artificial intelligence technologies in education as helpful for learning, relatively fair, and understandable and manageable, their levels of education anxiety tend to be lower, and their mental health tends to be more positive. This finding is consistent with the core propositions of control-value theory and stress–cognitive appraisal theory, which suggest that individuals' cognitive evaluations of situational value, the possibility of control, and environmental justice are closely related to their emotional experiences and psychological states (34). Particularly noteworthy is that, in this study, perceived fairness was found to be more important than perceived usefulness, indicating that in AI-supported educational contexts, students are concerned not only with whether the technology is “useful,” but also with whether it “affects them fairly.”

This finding has practical implications for public mental health policy and the governance of artificial intelligence in education. From the perspective of mental health promotion, students' anxiety in digital educational environments does not arise solely from academic burden itself; it may also stem from perceptions of opaque evaluation rules (35), unexplained system judgments (36), and uncertainty in the allocation of educational opportunities (37). Therefore, mental health interventions targeting university students should not remain limited to emotional counseling at the individual level, but should also pay attention to whether the educational technology environment itself can provide a sufficient sense of fairness, comprehensibility, and controllability. From the perspective of AI governance, the findings suggest that the design and deployment of educational AI should move beyond the singular logic of “efficiency first” and place greater emphasis on algorithmic transparency, the explainability of evaluation rules, appeal and feedback mechanisms, and students' perceptions of the boundaries of system control. In other words, enhancing the trustworthiness and procedural justice of educational AI may not only facilitate technology acceptance, but also contribute to fostering an educational environment with a stronger sense of psychological safety.

At the same time, the findings of this study should be interpreted within a specific cultural context. The sample in this study was drawn from Chinese university students, and the Chinese educational context has long been characterized by intense competition for academic advancement, a strong evaluation orientation, and salient collective norms. Against this background, students may be more sensitive to educational fairness, evaluation procedures, and opportunities for development; therefore, the strong association of perceived fairness within the research model is not surprising. By contrast, in countries or regions with different educational systems, cultural value orientations, and levels of student autonomy, the strength and mechanisms of the relationships between AI-related perceptions, education anxiety, and mental health may differ.

### 6.2 Research limitations and future prospects

This study still has several limitations that need to be addressed in future research. First, the data are based on cross-sectional self-report measures, which makes it difficult to fully identify causal directions and long-term effects, and may also be subject to common method bias. Although statistical tests can partially control for same-source bias, they cannot substitute for time-series evidence. Future studies could combine longitudinal tracking, cross-lagged panel models, and situational experiments or randomized controlled trials to further test whether “enhancing perceived fairness and controllability can sustainably reduce educational anxiety and improve mental health,” and to examine the stability and cumulative effects of this influence over different time spans. Second, the sample may be concentrated in specific regions, educational stages, or institutional contexts, which limits the generalizability of the findings. The meaning and mechanisms of perceived fairness and perceived controllability may vary across examination-oriented cultures, elite educational settings, and diversified assessment systems. Subsequent studies should conduct multi-site sampling across different regions, educational stages (e.g., primary, lower secondary, upper secondary, and higher education), and school types, and use multi-group SEM to test measurement invariance of the scales and structural paths. At the same time, cross-cultural comparisons could be conducted to examine how students in different educational systems understand technological fairness and controllability, and to delineate the boundary conditions of their effects on educational anxiety and mental health. Third, the theoretical model remains relatively narrow in terms of variable selection, including only technology-related perceptions, educational anxiety, and mental health, without incorporating important contextual factors such as teacher support, classroom climate, school climate, family expectations, and parenting styles, nor considering the moderating roles of individual traits such as self-efficacy, academic resilience, coping styles, and personality characteristics. Future research could, within an ecological systems theoretical framework, construct a multilevel integrative model of “individual–family–school–technology system,” apply multilevel modeling to examine cross-level effects of classroom- and school-level factors, and explore the moderating or mediating pathways of individual characteristics in the relationships among technology perceptions, educational anxiety, and mental health.

### 6.3 Conclusion

This study, based on a dual analysis using structural equation modeling and artificial neural networks, systematically examined the relationships among perceived usefulness, perceived fairness, perceived controllability, education anxiety, and student mental health. The results indicate that all three dimensions of technology perception are significantly associated with student mental health and are also related to lower levels of education anxiety. Among them, perceived fairness and perceived controllability showed relatively stronger associations in explaining variations in education anxiety and mental health. ANN sensitivity analysis further supported these findings, showing that perceived fairness had the highest importance in predicting education anxiety, followed by perceived controllability and perceived usefulness, thereby enhancing the consistency and robustness of the results at the methodological level.

Theoretically, this study integrates the frameworks of technology acceptance, control-value theory, and stress–cognitive appraisal, and develops a comprehensive analytical model of “technology perception–education anxiety–mental health,” thereby extending the perspective of student mental health research in the context of educational digitalization. Practically, the findings highlight the importance of the coordinated optimization of usefulness, fairness, and controllability in educational technology and educational governance. This suggests that, in advancing digital transformation in education and attending to student psychological wellbeing, greater emphasis should be placed on improving procedural and outcome fairness, strengthening students' sense of control over learning and evaluation processes, and ensuring that educational technology provides meaningful support for students' actual learning needs, so as to be more closely associated with lower levels of education anxiety and more positive mental health outcomes.

## Data Availability

The original contributions presented in the study are included in the article/supplementary material, further inquiries can be directed to the corresponding author.
